# Conversion Surgery for Pancreatic Cancer—The Impact of Neoadjuvant Treatment

**DOI:** 10.3389/fonc.2019.01501

**Published:** 2020-01-14

**Authors:** Ulla Klaiber, Thilo Hackert

**Affiliations:** Department of General, Visceral and Transplantation Surgery, University of Heidelberg, Heidelberg, Germany

**Keywords:** pancreatic cancer, neoadjuvant therapy, FOLFIRINOX, locally advanced pancreatic cancer, borderline resectable pancreatic cancer, conversion surgery

## Abstract

Pancreatic ductal adenocarcinoma (PDAC) has still a dismal prognosis, mainly because only 15–20% of all patients present with resectable tumor stages at the time of diagnosis. Due to locally extended tumor growth or distant metastases upfront resection is not reasonable in the majority of patients. Considerably, PDAC will be the 2nd most frequent cause of cancer-related deaths within the next 10 years for both men and women. While there is currently no convincing evidence for the use of neoadjuvant therapy in resectable PDAC, there are controversial results from studies investigating neoadjuvant treatment concepts in borderline resectable PDAC (BR-PDAC). However, the definition of BR-PDAC is a topic of debate. While BR-PDAC has originally been defined on merely anatomical criteria, the International Association of Pancreatology (IAP) has recently suggested a broader definition based on a combination of anatomical (A) findings, biological (B) criteria (which reflect tumor aggressiveness), and conditional (C) aspects (which respect host-related condition). In case of BR-PDAC with venous invasion alone, upfront resection is generally recommended whenever technically possible in patients fit for surgery and without evidence for lymph node metastases. In contrast, in case of arterial invasion neoadjuvant therapy is regarded as the treatment of choice. The same accounts for high CA 19-9 levels, suspected or proven lymph node involvement and poor performance status. In locally advanced PDAC (LA-PDAC), neoadjuvant treatment represents the standard of care resulting in proportionally high rates of secondary resection. This “conversion” surgery offers the chance for improved survival times in an otherwise palliative situation. Herein, we summarize the current evidence of different treatment strategies for pancreatic cancer with a focus on conversion surgery and the impact of neoadjuvant treatment in this setting.

## Background

Pancreatic ductal adenocarcinoma (PDAC) is one of the most lethal tumor entities and—although being currently the 3rd leading cause for cancer-associated mortality in the United States—will be the 2nd leading cause of cancer-related death within the next 10 years. This frustrating development can mainly be explained by an increasing incidence and still limited treatment success (1, 2). Only about 15–20% of all patients diagnosed with PDAC qualify for upfront resection, while in the majority of patients, locally extended tumor growth, or distant tumor spread are found at diagnosis (3). Over the last 10 years, the term “borderline” resectable PDAC (BR-PDAC) has been developed to better characterize the subgroup of patients with a locally more extended, but still technically resectable tumor. Today, different definitions of resectability are used. Firstly, the joint consensus guideline of the Americas Hepato-Pancreato-Biliary Association (AHPBA), the Society of Surgical Oncology (SSO) and the Society for Surgery of the Alimentary Tract (SSAT) (4), and secondly, the definition of the International Study Group of Pancreatic Surgery (ISGPS) (5), which mainly refers to the resectability definitions of the National Comprehensive Cancer Network (NCCN) (6). According to these definitions, the extension of the primary tumor is generally classified as (1) resectable, (2) borderline resectable (BR-PDAC), or (3) locally advanced (LA-PDAC), with slight differences depending on the classification system used. In this review article, we exclusively refer to the ISGPS definition (5). In case of distant metastases, the tumor basically cannot be assigned to any of these three groups, as stage IV PDAC has traditionally been considered as a palliative situation—regardless of the local tumor extension. However, several recent studies showed that conversion surgery including resection of single metastases after neoadjuvant treatment may have an oncological benefit in highly selected patients with oligometastatic disease (7, 8).

**Figure 1:**
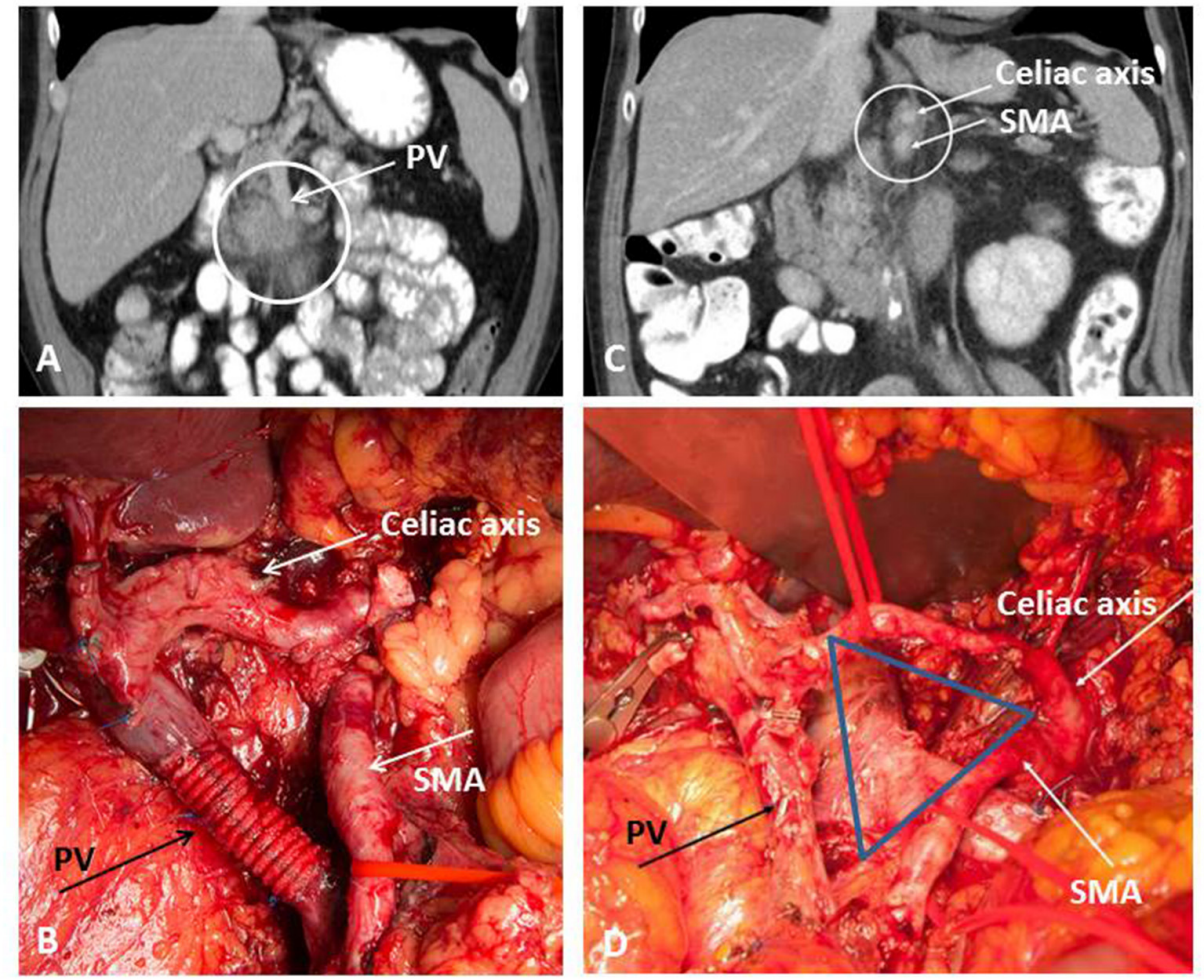
Extended pancreatic resection for BR-PDAC **(A,B)** and conversion surgery for LA-PDAC **(C,D)**. **(A)** Preoperative imaging demonstrating involvement of the portal vein (PV); **(B)** operative site after extended tumor resection with type 4 resection of the PV; **(C)** partial response on restaging after Folfirinox; **(D)** operative site after pancreatectomy with artery first approach and TRIANGLE-procedure comprising radical dissection directly on the arterial wall of the superior mesenteric artery (SMA) and the celiac axis (CA).

From the anatomical point of view, resectable PDAC is defined as a locally limited tumor extension without any tumor contact with the superior mesenteric (SMV) or portal vein (PV), and also without involvement of the celiac axis (CA), its main branches and the superior mesenteric artery (SMA). In case of venous involvement, the criteria for BR-PDAC are fulfilled if venous resection followed by reconstruction is technically feasible. Cavernous collateralization of the portal venous axis toward the hepatic hilus or infiltration of the jejunal vein branches are findings of venous tumor involvement without the possibility of venous reconstruction and thereby fulfill the criteria of LA-PDAC, with the particularity that conversion surgery is rarely possible in these two scenarios despite neoadjuvant treatment. Arterial BR-PDAC is defined as an abutment below 180° of the SMA or isolated tumor contact to the hepatic artery. In addition to the situations described above, LA-PDAC comprises tumors with a more extended involvement of the SMA, CA, common hepatic artery, aorta, or inferior vena cava (9). Figure 1 shows one typical case of venous BR-PDAC and another case with LA-PDAC.

Besides the aspect of technical resectability, tumor biology must be considered independently from local tumor extension as there are patients who may have a localized and resectable, but biologically very aggressive tumor which implies that they have a high risk of local tumor recurrence or metastases very early after surgery. Unfortunately, today the diagnostic tools to preoperatively characterize the aggressiveness of the individual patient's tumor are highly limited as no precise markers have been available so far. Since years, the best clinically established marker for PDAC has been carbohydrate antigen (CA) 19-9 which gives a rough estimate on tumor biology although cut-off values to differentiate highly aggressive from moderate or low aggressive tumors are rather unreliable. In addition, ~15% of all patients do not express CA 19-9 at all, and in case of obstructive jaundice the interpretation of the CA 19-9 level is also very limited. The International Association of Pancreatology (IAP) was the first to consider tumor biology in their current definition of borderline resectability which was published 2 years ago (10). Besides the anatomical criteria as already defined, the IAP definition also comprises biological and conditional parameters to determine resectability. Accordingly, a locally resectable tumor may be categorized as BR-PDAC as soon as considerably increased CA 19-9 levels (i.e., >500 units/ml) or regional lymph node metastases (i.e., biopsy proven or highly suspected on PET/CT scan) are found, and/or if the individual patient's clinical condition is significantly compromised (i.e., reduced performance status)—making the completion of adjuvant therapy after major pancreatic surgery unlikely. The rationale for this classification system is that the oncological outcome is not only determinated by local resectability and operatively influencable factors like resection margin status, but tumor biology and patient condition also play a decisive role (10).

## Indications for Upfront Surgery

Patients fit for surgery with a resectable tumor should undergo surgical exploration and radical resection followed by adjuvant chemotherapy as long as convincing evidence for neoadjuvant treatment in this situation is lacking (9). Consequently, neoadjuvant therapy for resectable PDAC should only be performed after enrolment of the individual patient in a clinical trial, preferably within a randomized trial design (11, 12). Based on the existing evidence, patients with BR-PDAC should be treated depending on the type of vascular involvement. In case of venous BR-PDAC complete tumor removal combined with venous replacement is the treatment of choice (Figure 1B), while in arterial BR-PDAC the decision for upfront resection must be made more critically due to reduced rates of R0 resections, among other aspects (11, 12).

## Neoadjuvant Treatment in Borderline Resectable Pancreatic Cancer (BR-PDAC)

Neoadjuvant treatment followed by conversion surgery represents a potentially beneficial treatment alternative for BR-PDAC and is currently evaluated in numerous studies including patients with either type of BR-PDAC (i.e., venous type, arterial type, IAP type B, or C) (11, 12). Most of the available evidence has been retrieved from retrospective studies and registry analyses, however, meanwhile the first results from randomized controlled trials (RCTs) have been published. The main shortcomings of the existing retrospective studies on neoadjuvant treatment in BR-PDAC are low sample sizes in each study and considerable heterogeneity in chemotherapy and chemoradiotherapy protocols, making inter-study comparisons difficult (13–16). About 50% of all patients included in these studies showed a stable tumor growth after neoadjuvant treatment, whereas a partial tumor response was observed in 30%, and in only 3% of the patients a complete response occurred (13). One out of five patients developed tumor progression during neoadjuvant therapy, illustrating the current problem of non-predictable tumor response to a specific chemotherapy protocol. This aspect is of particular importance because it either shows the selection effect of patients suffering from a generally unfavorable tumor biology or it may show that patients simply received the “wrong” therapy, underlining the urgent need for precision oncology in the fight against PDAC. In patients with stable disease or (partial) response after neoadjuvant therapy, a tumor resection was possible in two out of three patients, including R0 resections in ~60% of patients and a median survival time of 26 months, which is comparable to the oncological outcome after upfront resection (13). In a recently published multicenter RCT from Korea, 27 patients received neoadjuvant therapy (i.e., chemoradiation with gemcitabine and 54 Gy) and 23 patients underwent upfront surgery (17). The study was terminated prematurely due to a clear survival benefit of the neoadjuvant treatment arm. The Dutch randomized controlled PREOPANC trial was designed to allocate patients with resectable and BR-PDAC to either neoadjuvant chemoradiotherapy or upfront resection (18). One hundred and twenty-seven patients with upfront resection were compared to 119 patients with neoadjuvant therapy, whereas in both groups ~50% of the patients were classified as BR-PDAC. The study failed to demonstrate a significant benefit of neoadjuvant therapy in terms of overall survival and showed rather disappointing results of 14 (upfront surgery group) vs. 17 months (neoadjuvant therapy group) of median survival.

These two examples of RCTs show two aspects that make a valid conclusion on the actual value of neoadjuvant therapy for BR-PDAC very difficult. First, most of the current studies have included very heterogeneous patient populations (i.e., resectable and BR-PDAC or BR- and LA-PDAC). Second, there is no international standard on neoadjuvant protocols at the moment so that institutions around the world chose their preferred treatment including chemotherapy, chemoradiation, or a sequential treatment pre- and postoperatively with both modalities. In contrast to this, the standards of adjuvant therapy after upfront resection are clearly defined on the basis of large multicenter studies making this concept an evidence-based approach.

## Neoadjuvant Therapy in Locally Advanced Pancreatic Cancer (LA-PDAC) and Conversion Surgery

Regarding LA-PDAC, neoadjuvant therapy, and conversion surgery have undergone a considerable development in recent years. While venous LA-PDAC is characterized by a technically unreconstructable venous tract, arterial LA-PDAC is basically technically resectable but in general considered as unfavorable from an oncological point of view as it may reflect an extremely aggressive tumor biology. Consequently, venous LA-PDAC is unlikely to become technically resectable after neoadjuvant treatment, whereas arterial LA-PDAC has to be regarded differently.

In this context, tumors of the pancreatic body which involve the CA represent a special situation in which a distal pancreatectomy with transection of the CA and the hepatic artery under preservation of the gastroduodenal artery can be performed if the arterial perfusion of the liver is ensured by retrograde blood from the SMA (so-called DP-CAR, modified Appleby procedure). Preferably, DP-CAR is embedded in a neoadjuvant setting as shown in most recent studies. A cohort study from Johns Hopkins included 17 patients with PDAC of the pancreatic body undergoing DP-CAR following neoadjuvant treatment and matched them with 51 patients undergoing DP only (19). In the DP-CAR group, duration of operation was prolonged and postoperative liver enzymes were elevated, whereas no differences were observed with regard to intraoperative blood loss, postoperative length of hospital stay, and microscopic tumor clearance. The median survival times did not differ significantly between both groups (DP-CAR: 20 vs. DP only: 19 months) (19). A multicenter study from Japan reported on 72 patients undergoing DP-CAR with a neoadjuvant therapy in 56% of all patients (20). In this study, median survival was 18 months and adjuvant therapy was found to be a significant prognostic factor in the DP-CAR collective (20). The results from these studies were confirmed by a systematic review, including 19 studies with a total number of 240 patients, which showed that, despite a considerably high morbidity, DP-CAR can be performed with a relatively low mortality of 3.5% and results in 15 months of median survival—which can further be improved when resection is embedded in a multimodal approach including neoadjuvant therapy (21). A worldwide survey included real-world data from 23 centers with 191 patients undergoing DP-CAR (22). With a 90-day mortality of 5.5% in high-volume and 18% in low-volume centers, the risk score analysis revealed that international practice for DP-CAR is not standardized (i.e., type of preoperative therapy or CA embolization) and that patient selection and center expertise are the most important factors to achieve beneficial results.

Besides the above mentioned scenario allowing DP-CAR, arterial LA-PDAC has historically been regarded as a palliative situation and arterial resections in PDAC surgery have only been performed in few patients in the past (23, 24). This can mainly be explained by increased rates of postoperative complications and mortality which is particularly high when arterial resection is performed during partial pancreatic resection bearing the risk of severe pancreatic fistula and post-pancreatectomy hemorrhage. In addition, oncologic outcomes reported in the literature addressing arterial resection during PDAC surgery have been disappointing (25, 26). A systematic review and meta-analysis showed inferior 1- and 3-year survival rates in 170 patients undergoing pancreatic resection with arterial resection compared to 1,640 patients undergoing standard resection (25). Another systematic review focusing specifically on the resection of the SMA during pancreatic resection included 13 retrospective studies with a total of 70 patients (26). Unacceptably high morbidity and mortality rates were confirmed by this study (mortality in 20% of patients) and median survival was only 11 months (26), which is not superior to a palliative treatment with modern chemotherapy protocols (27). Consequently, upfront resection in LA-PDAC is in general not justified from an oncologic point of view nor technically feasible in most cases. As reported in two recent case series from highly experienced centers, however, upfront resection may be performed as an individual approach in very carefully selected patients (28, 29). In these two series, 14 and 34 patients, respectively, underwent PDAC surgery including arterial resection which was associated with very low mortality (0 and 3%, respectively) and good oncological results (up to 42% 5-year survival).

Modern neoadjuvant therapy has changed the picture of arterial resection being an only highly-individual approach and it has been shown that effective neoadjuvant protocols can considerably increase secondary resection rates despite an unresectable tumor stage at the time of diagnosis. This development can be referred to two main potential effects of neoadjuvant therapy: (1) down-sizing and maybe down-staging of the tumor, and (2) devitalizing the tumor without an obvious imaging-based response which allows to spare arterial resections in many patients during conversion surgery. The Japanese Society of Hepato-Biliary-Pancreatic Surgery published a series including 58 patients with initially unresectable PDAC who underwent 8 months of neoadjuvant treatment followed by “adjuvant resection” which resulted in clearly improved survival times compared to a palliative approach, illustrating the important role of neoadjuvant treatment in conversion surgery (30).

## Evaluation of Tumor Response During Neoadjuvant Therapy

The evaluation of tumor response to neoadjuvant therapy remains an unsolved problem in the treatment of LA-PDAC because a clearly radiographic response can be seen in only approximate one third of the patients (31). There is growing consensus that conventional imaging by standard contrast-enhanced computed tomography (CE-CT) fails to reflect tumor response to neoadjuvant therapy (32). This has been demonstrated by a series including 50 patients with BR-PDAC or LA-PDAC in whom conversion surgery was successfully performed in more than 50% of patients after neoadjuvant treatment despite no significant imaging-based changes during restating (32). In this study, median survival was 23 months after resection compared to 13 months in unresected patients, indicating the prognostic impact of tumor removal on survival in BR-PDAC and LA-PDAC patients (32). This was confirmed by many other authors in the era of FOLFIRINOX. In a study including 40 patients, among which 26 patients suffered LA-PDAC, R0 resection rate after neoadjuvant treatment was 92% although a radiographic response was not seen in most patients during restaging (33). A pathologic downstaging of the tumor was clearly observed on the final pathology resulting in a decreased proportion of patients with positive lymph nodes when compared with patients undergoing upfront resection (35 vs. 79%), less lymphatic invasion (35 vs. 70%) and reduced perineural invasion (73 vs. 95%) (33). A large series from Heidelberg included 575 patients undergoing neoadjuvant treatment for LA-PDAC (34). Three hundred and twenty-two patients received gemcitabine and radiotherapy, 125 patients received FOLFIRINOX, and 128 patients were treated on the basis of other protocols (excluding FOLFIRINOX). The majority of patients' tumors were staged as locally advanced due to arterial involvement or evidence for distant tumor spread at the time of diagnosis. All patients included in this study underwent exploration after the neoadjuvant treatment period and restaging. In 292 of 575 patients (50.8%) a successful resection was performed. The resection rates differed significantly between the treatment groups with 61% (76 of 125 patients) after FOLFIRINOX, compared with 46% (150 of 322 patients) after gemcitabine and radiation, and 52% (66 of 128 patients) after other treatments (*P* = 0.026). The median overall survival after resection was 15.3 months compared with 8.5 months after exploration alone (*P* < 0.0001). When considering the neoadjuvant treatment period, further 5–6 months of survival are to be added (34). The Heidelberg policy is to offer exploration to all patients who present with stable disease or even remission after neoadjuvant treatment, while patients with progressive disease or worsened clinical condition continue systemic treatment. Successful conversion surgery can be performed after intraoperative exclusion of distant metastases and evaluation of the critical arteries, i.e., the SMA and CA (artery-first approach) (35). In patients with good response to neoadjuvant treatment, frozen section histology often does not show any viable tumor surrounding these arteries allowing tumor removal without vascular resection. In this situation, a periarterial dissection with radical removal of all soft tissue between the origin of the CA and the SMA and the mesenterico-portal venous axis should be performed to increase the chance of an R0 resection. As described by Hackert et al., this “TRIANGLE” procedure (Figure 1D) can be performed from the right, the left or both sides, depending on the site of the tumor and the intraoperative findings (36). In many cases, a synchronous venous resection might be necessary to ensure complete tumor clearance. The TRIANGLE procedure is comparable to the previously described systematic meso-pancreas dissection using a supracolic anterior artery-first approach in upfront pancreatoduodenectomy (37). Compared to upfront resection, prognostic factors after conversion surgery include lymph node status, vascular involvement, presence of distant metastases, and CA 19-9 levels (38).

## Future Perspectives

Recent meta-analyses on the efficacy of neoadjuvant treatment in patients with LA-PDAC confirmed the benefit of pretreatment, primarily with FOLFIRINOX, but also revealed low evidence for the use of neoadjuvant chemotherapy and chemoradiotherapy owing to the limited methodological quality of the original publications available (39, 40). One of the shortcomings of the primary studies is the mostly retrospective study design as well as the inclusion of heterogenous study populations, for instance both BR-PDAC and LA-PDAC patients which makes intra- and inter-study comparisons difficult. Considering this lack of evidence, results from high-quality randomized trials are urgently needed to optimize the oncosurgical management of pancreatic cancer. One of the aims of such trials should be to evaluate the benefit of radiation and to define the most effective chemotherapy protocols and the optimal sequence as well as duration of the neoadjuvant treatment period.

Considering the limited suitability of conventional imaging, diagnostic tools to better evaluate response to neoadjuvant treatment would be desirable as well as biomarkers to better select patients who will benefit from a multimodal treatment approach. Although no clear cut-off values for CA 19-9 are defined, however, the relative drop of CA 19-9 after neoadjuvant therapy can give additional information on potential resectability and oncological prognosis (41, 42). In this context, better criteria to select patients for metastases resection and arterial resection during conversion surgery are also needed.

## Summary

In summary, the paradigm of regarding LA-PDAC as a non-surgical and palliative disease is undergoing substantial changes today. Different approaches of neoadjuvant therapy show encouraging results underlining the importance of a multimodal strategy to improve these patients' prognosis. Standardization of neoadjuvant approaches, however, remains poor to date. Still the aim of conversion surgery in LA-PDAC can be achieved in a considerable number of patients and effective chemotherapy protocols with or without radiation are the key to further enhance these results and should be investigated in prospective clinical trials to allow evidence-based recommendations in the future.

## Author Contributions

TH and UK conceived and wrote this review article.

### Conflict of Interest

The authors declare that the research was conducted in the absence of any commercial or financial relationships that could be construed as a potential conflict of interest.
